# Doctors and Drug Supply

**Published:** 1904-02-13

**Authors:** 


### The Hospital.

A Journal of

The Medical Sciences and Hospital Administration.

Vol. XXXV.—No. 907. FEBRUARY 13, 1904.

### Doctors and Drug Supply.

A statement has been publicly made, apparently upon some sort of authority, that an intention is seriously entertained, among a certain section of so-called medical reformers, of asking the legislature of this country to pass an enactment by which it would be rendered a penal offence for a registered medical practitioner to supply medicine to his patients. It is well known that suggestions for what is humorously described as the "amendment" of the existing Medical Acts have for some time been floating in the air; and that to which we refer is said to be the latest addition to them. It is, we are told, if possible, to be incorporated with the programme of the reformers at a forthcoming meeting of the Metropolitan Counties' Branch of the British Medical Association, where it is to be duly proposed, seconded, and voted upon; and it suggests a penalty of £100 (we have not seen any reference to the corresponding period of imprisonment in default of payment) for the "first offence," and for the second removal from the Medical Register, we presume as an official act by the General Medical Council, based upon the record of conviction, as at present in the case of felony or misdemeanour. Exception is to be made in the case of medical men holding certain public offices, and also in the case of those practising in districts not sufficiently populated to be able to support both a doctor and a drug seller, although the question of the standards by which such ability should be estimated might open the door to a good deal of controversy in any particular instance.

The general reputation of the medical profession for common sense is an asset of great value in all its dealings with the public; and, for the sake of that reputation, we trust that the proposal to which we have referred will be promptly snuffed out by the Metropolitan or any other branch of the association before which it may be brought for consideration. The idea that the British Legislature would ever render it a penal offence for any man, whether medical practitioner or not, who was honestly in possession of a box of pills, to sell those pills to anyone who desired to purchase them, is too childishly absurd for serious consideration; but the proposal to do so, if it were definitely made on the part of the medical profession, would cover that profession with ridicule, the effects of which would not be outlived for years. Every proposal for better legislation with regard to sanitary control, to the restraint of quackery, to the better registration of deaths, or to any other subject recommended to Parliament by a consensus of medical opinion, would be met by the scornful outcry that these were the people who had wished to fine a man a hundred pounds for selling a box of pills, and to deprive him of his professional status and his means of livelihood if he sold half the contents of the box upon one occasion, and the remainder upon another. The profession cannot afford to have such "fantastic tricks" played in its name, or by those who profess to speak with its authority.

It is impossible to deny, nevertheless, that a separation between the functions of the doctor and of the drug seller would be an end well worth striving for by legitimate means; more especially if it could be so accomplished as to bring back the day of individual prescribing for patients, together with a general recognition of the fact that medicines are disturbing elements, not to be introduced into the economy without grave and sufficient reason. We have arrived, during the last few years, at a state of public opinion in which ignorant persons are in the habit of dosing themselves with poisons with as little compunction as our grandparents felt about eating rumpsteaks and drinking old October; and we are supposed by a great many people to be, as a nation, lapsing into physical degeneracy. A New York journal has lately called attention to the large reported increase of deaths from "heart failure" among middle-aged people since they have taken to treating themselves for "headache" with phenacetine and other such compounds, of the action of which they know nothing, and the medical profession itself not quite so much as might be desired. An enormous educational gain would arise if it were impossible to buy active medicines without a medical prescription for definite quantities definitely compounded; and if such a prescription could not be repeated without the authority of the prescriber. Such an idea, in the present state of civilisation, is probably Utopian; but the proper way of separating the compounder from the prescriber of medicine would be by the action of the Royal College of Physicians, not by that of the legislature. The College, in succession to the Society of Apothecaries, has become the chief source of the licenses of general practitioners in England, and it is open to the College to frame any reasonable regulations subject to which its license shall be held. It might forbid its licentiates to supply medicine, except under conditions satisfactory to the Censors Board, and by special permission thereof. Year by year, the Censors Board might require clearer evidence of necessity before the permission was granted; and, in such case, the result would be that the detachment of doctors from physic would be gradually rendered more complete, and the public would be gradually educated to the knowledge that drugs form only part, and often not the most important part, of the medical armamentarium. It would be possible, at the same time, for the college to set its face steadily against all those so-called "improvements" of modern pharmacy of which the first result is to substitute the manufacturer for the practitioner as a prescriber, and the second to assist the patient himself in playing with edge-tools of the nature of which he is entirely ignorant. The medical profession itself is, we believe, very largely responsible for the plague of drugs which of late years has swept over the country, and it might fairly be called upon to bestir itself in any manner by which there was a prospect of the plague being stayed.

